# Supplementary material for: Are children and dogs best friends? A scoping review to explore the positive and negative effects of child-dog interactions
Source: PeerJ. 2022 Dec 19;10:e14532. doi: 10.7717/peerj.14532 (PMC9774011; doi:10.7717/peerj.14532)
Supplement: Supplemental Information 1 [file peerj-10-14532-s001.docx]

# Are children and dogs best friends? A scoping review to explore the positive and negative effects of child-dog interactions

Claire S. E. Giraudet, Kai Liu, Alan G. McElligott, and Mia Cobb

Appendix

This appendix gives the list of the 317 reviewed sources (in alphabetical order according to first author) found after searching the SCOPUS database, filtering for key inclusion criteria, and removing duplicates.

Figure 1, Figure 2, and Figure 3 are based on the information provided in this table.

| **Authors** | **Year of publication** | **Title** | **Journal** | **Doi** | **Type of document** | **Country** |
| --- | --- | --- | --- | --- | --- | --- |
| Abadi *et al.* | 2022 | Dog-assisted physical activity intervention in children with autism spectrum disorder: a feasibility and efficacy exploratory study | Anthrozoos | 10.1080/08927936.2022.2027091 | Article | Canada |
| Aldridge *et al.* | 2019 | Young children's interpretation of dogs' emotions and their intentions to approach happy, angry, and frightened dogs | Anthrozoos | 10.1080/08927936.2019.1598656 | Article | UK |
| Amparo *et al.* | 2019 | Impact of integrating rabies education into the curriculum of public elementary schools in Ilocos Norte, Philippines on rabies knowledge, and animal bite incidence | Frontiers in Public Health | 10.3389/fpubh.2019.00119 | Article | Philippines |
| Anyo *et al.* | 2002 | Early, current and past pet ownership: associations with sensitization, bronchial responsiveness and allergic symptoms in school children | Clinical and Experimental Allergy | 10.1046/j.1365-2222.2002.01254.x | Article | Netherlands |
| Apfelbacher *et al.* | 2010 | Contact to cat or dog, allergies and parental education | Pediatric Allergy and Immunology | 10.1111/j.1399-3038.2009.00893.x | Article | Germany |

| **Authors** | **Year of publication** | **Title** | **Journal** | **Doi** | **Type of document** | **Country** |
| --- | --- | --- | --- | --- | --- | --- |
| Arai *et al.* | 2011 | Importance of bringing dogs in contact with children during their socialization period for better behavior | Journal of Veterinary Medical Science | 10.1292/jvms.10-0445 | Article | Japan |
| Arhant *et al.* | 2016 | Attitudes of caregivers to supervision of child family dog interactions in children up to 6 years” An exploratory study | Journal of Veterinary Behavior: Clinical Applications and Research | 10.1016/j.jveb.2016.06.007 | Article | Several |
| Arhant *et al.* | 2017 | Caregiver reports of interactions between children up to 6 years and their family dog-implications for dog bite prevention | Frontiers in Veterinary Science | 10.3389/fvets.2017.00130 | Article | Several |
| Asarnoj *et al.* | 2016 | Sensitization to cat and dog allergen molecules in childhood and prediction of symptoms of cat and dog allergy in adolescence: a BAMSE/MeDALL study | Journal of Allergy and Clinical Immunology | 10.1016/j.jaci.2015.09.052 | Article | Sweden |

| **Authors** | **Year of publication** | **Title** | **Journal** | **Doi** | **Type of document** | **Country** |
| --- | --- | --- | --- | --- | --- | --- |
| Auplish *et al.* | 2017 | Estimating the intra-cluster correlation coefficient for evaluating an educational intervention program to improve rabies awareness and dog bite prevention among children in Sikkim, India: a pilot study | Acta Tropica | 10.1016/j.actatropica.2016.12.032 | Article | India |
| Ávila-Álvarez *et al.* | 2020 | Assessing the outcomes of an animal-assisted intervention in a paediatric day hospital: perceptions of children and parents | Animals | 10.3390/ani10101788 | Article | Spain |
| Avner *et al.* | 1991 | Dog bites in urban children | NA | 10.1542/peds.88.1.55 | Conference paper | USA |
| Baatz *et al.* | 2020 | Education as a tool for improving canine welfare: evaluating the effect of an education workshop on attitudes to responsible dog ownership and canine welfare in a sample of Key Stage 2 children in the United Kingdom | PLoS ONE | 10.1371/journal.pone.0230832 | Article | UK |

| **Authors** | **Year of publication** | **Title** | **Journal** | **Doi** | **Type of document** | **Country** |
| --- | --- | --- | --- | --- | --- | --- |
| Beck *et al.* | 1985 | Unreported dog bites in children | Public Health Reports | NA | Article | USA |
| Bedolla-Barajas *et al.* | 2018 | Exposure to dogs but not cats is associated to a decrease in the prevalence in atopic dermatitis amongst school-children | Allergologia et Immunopathologia | 10.1016/j.aller.2017.09.032 | Article | Mexico |
| Beetz | 2013 | Socio-emotional correlates of a schooldog-teacher-team in the classroom | Frontiers in Psychology | 10.3389/fpsyg.2013.00886 | Article | USA |
| Beetz *et al.* | 2011 | The effect of a real dog, toy dog and friendly person on insecurely attached children during a stressful task: an exploratory study | Anthrozoos | 10.2752/175303711X13159027359746 | Article | Several |
| Ben-Itzchak *et al.* | 2021 | Dog training intervention improves adaptive social communication skills in young children with autism spectrum disorder: A controlled crossover study | Autism | 10.1177/13623613211000501 | Article | Isreal |
| Bergman *et al.* | 2008 | Expanding families: preparing for and introducing dogs and cats to infants, children, and new pets | Veterinary Clinics of North America - Small Animal Practice | 10.1016/j.cvsm.2008.04.004 | Review | World |

| **Authors** | **Year of publication** | **Title** | **Journal** | **Doi** | **Type of document** | **Country** |
| --- | --- | --- | --- | --- | --- | --- |
| Bernardo *et al.* | 1998 | Dog bites in children admitted to Pennsylvania trauma centers | International Journal of Trauma Nursing | 10.1016/S1075-4210(98)90001-3 | Article | USA |
| Bernardo *et al.* | 2000 | Dog bites in children treated in a pediatric emergency department | Journal of the Society of Pediatric Nurses | 10.1111/j.1744-6155.2000.tb00090.x | Article | USA |
| Bernardo *et al.* | 2002 | A comparison of dog bite injuries in younger and older children treated in a pediatric emergency department | Pediatric Emergency Care | 10.1097/00006565-200206000-00024 | Article | USA |
| Berry *et al.* | 2013 | Use of assistance and therapy dogs for children with autism spectrum disorders: A critical review of the current evidence | Journal of Alternative and Complementary Medicine | 10.1089/acm.2011.0835 | Review | World |
| Bharti *et al.* | 2019 | Failure of postexposure prophylaxis in a girl child attacked by rabid dog severing her facial nerve causing possible direct entry of rabies virus into the facial nerve | Human Vaccines and Immunotherapeutics | 10.1080/21645515.2019.1608131 | Article | India |
| Bjork *et al.* | 2013 | Dog bite injuries among American Indian and Alaska native children | Journal of Pediatrics | 10.1016/j.jpeds.2012.11.087 | Article | USA |
| Black *et al.* | 1996 | Dog bites in children | Plastic Surgical Nursing | NA | Review | World |

| **Authors** | **Year of publication** | **Title** | **Journal** | **Doi** | **Type of document** | **Country** |
| --- | --- | --- | --- | --- | --- | --- |
| Blouin | 2013 | Are dogs children, companions, or just animals? Understanding variations in people's orientations toward animals | Anthrozoos | 10.2752/175303713X13636846944402 | Review | World |
| Boat | 2019 | Dog bites to children: family interventions and prevention strategies | NA | 10.1016/B978-0-12-812962-3.00003-4 | Book chapter | World |
| Boenning *et al.* | 1983 | Dog bites in children: epidemiology, microbiology, and penicillin prophylactic therapy | American Journal of Emergency Medicine | 10.1016/0735-6757(83)90032-3 | Article | USA |
| Boglioli *et al.* | 2000 | Unusual infant death: dog attack or postmortem mutilation after child abuse? | American Journal of Forensic Medicine and Pathology | 10.1097/00000433-200012000-00019 | Article | USA |
| Boisvert *et al.* | 2021 | Animal-assisted play: a strategy for promoting children’s physical activity play | International Journal of Playwork Practice | 10.25035/ijpp.02.01.06 | Review | World |
| Borgi *et al.* | 2015 | Attitudes toward animals among kindergarten children: species preferences | Anthrozoos | 10.2752/089279315X14129350721939 | Article | Italy |
| Bothra *et al.* | 2011 | Dog bite injuries of genitalia in male infant and children | Urology Annals | 10.4103/0974-7796.84949 | Article | India |

| **Authors** | **Year of publication** | **Title** | **Journal** | **Doi** | **Type of document** | **Country** |
| --- | --- | --- | --- | --- | --- | --- |
| Bowman | 2018 | Final gifts: lessons children can learn from dogs about end-of-life, loss, and grief | NA | NA | Book chapter | World |
| Bråbäck *et al.* | 2001 | Atopy among schoolchildren in Northern and Southern Sweden in relation to pet ownership and early life events | Pediatric Allergy and Immunology | 10.1034/j.1399-3038.2001.012001004.x | Article | Sweden |
| Bratton *et al.* | 2018 | Ophthalmic manifestations of facial dog bites in children | Ophthalmic Plastic and Reconstructive Surgery | 10.1097/IOP.0000000000000875 | Article | USA |
| Brelsford *et al.* | 2017 | Animal-assisted interventions in the classroom: a systematic review | International Journal of Environmental Research and Public Health | 10.3390/ijerph14070669 | Review | World |
| Brogan *et al.* | 1995 | Severe dog bites in children | Pediatrics | NA | Article | USA |
| Brown *et al.* | 2001 | Vacuum-assisted closure in the treatment of a 9-year-old child with severe and multiple dog bite injuries of the thorax | Annals of Thoracic Surgery | 10.1016/s0003-4975(01)02844-2 | Article | UK |
| Bufford *et al.* | 2008 | Effects of dog ownership in early childhood on immune development and atopic diseases | Clinical and Experimental Allergy | 10.1111/j.1365-2222.2008.03018.x | Article | USA |

| **Authors** | **Year of publication** | **Title** | **Journal** | **Doi** | **Type of document** | **Country** |
| --- | --- | --- | --- | --- | --- | --- |
| Burrows *et al.* | 2008 | Factors affecting behavior and welfare of service dogs for children with autism spectrum disorder | Journal of Applied Animal Welfare Science | 10.1080/10888700701555550 | Article | Canada |
| Burrows *et al.* | 2008 | Sentinels of safety: service dogs ensure safety and enhance freedom and well-being for families with autistic children | Qualitative Health Research | 10.1177/1049732308327088 | Article | Canada |
| Burrows *et al.* | 2008 | Challenges of service-dog ownership for families with autistic children: lessons for veterinary practitioners | Journal of Veterinary Medical Education | 10.3138/jvme.35.4.559 | Article | Canada |
| Byrne *et al.* | 2016 | An examination of harm beliefs in dog fearful children | *Journal of Experimental Psychopathology* | 10.5127/jep.048015 | Article | UK |
| Caffrey *et al.* | 2019 | Insights about the epidemiology of dog bites in a Canadian city using a dog aggression scale and administrative data | Animals | 10.3390/ani9060324 | Article | Canada |
| Carlisle | 2014 | Pet dog ownership decisions for parents of children with autism spectrum disorder | Journal of Pediatric Nursing | 10.1016/j.pedn.2013.09.005 | Article | USA |

| **Authors** | **Year of publication** | **Title** | **Journal** | **Doi** | **Type of document** | **Country** |
| --- | --- | --- | --- | --- | --- | --- |
| Carlisle | 2015 | The social skills and attachment to dogs of children with autism spectrum disorder | Journal of Autism and Developmental Disorders | 10.1007/s10803-014-2267-7 | Article | USA |
| Cataldi *et al.* | 2011 | Dog attack resulting in evisceration in an infant | Pediatric Emergency Care | 10.1097/PEC.0b013e3182131fc7 | Article | USA |
| Cavalcanti *et al.* | 2017 | Facial dog bite injuries in children: a case report | International Journal of Surgery Case Reports | 10.1016/j.ijscr.2017.10.008 | Article | Brazil |
| Chapman *et al.* | 2000 | Preventing dog bites in children: randomised controlled trial of an educational intervention | British Medical Journal | 10.1136/bmj.320.7248.1512 | Article | Australia |
| Chen *et al.* | 2008 | Dog ownership and contact during childhood and later allergy development | European Respiratory Journal | 10.1183/09031936.00092807 | Article | Germany |
| Chen *et al.* | 2016 | A comparative study of dog-and cat-induced injury on incidence and risk factors among children | International Journal of Environmental Research and Public Health | 10.3390/ijerph13111079 | Article | China |
| Chen *et al.* | 2018 | Dog bite and injury awareness and prevention in migrant and left-behind children in China | Scientific Reports | 10.1038/s41598-018-34428-1 | Article | China |

| **Authors** | **Year of publication** | **Title** | **Journal** | **Doi** | **Type of document** | **Country** |
| --- | --- | --- | --- | --- | --- | --- |
| Chiam *et al.* | 2014 | Retrospective review of dog bite injuries in children presenting to a South Australian tertiary children's hospital emergency department | Journal of Paediatrics and Child Health | 10.1111/jpc.12642 | Review | Australia |
| Chooramani *et al.* | 1993 | Dog bite of the prolapsed rectum in a child | Pediatric Surgery International | 10.1007/BF02353016 | Article | India |
| Christian *et al.* | 2013 | Understanding the relationship between dog ownership and children's physical activity and sedentary behaviour | Pediatric Obesity | 10.1111/j.2047-6310.2012.00113.x | Article | Australia |
| Christian *et al.* | 2014 | Dog walking is associated with more outdoor play and independent mobility for children | Preventive Medicine | 10.1016/j.ypmed.2014.08.002 | Article | Australia |
| Christian *et al.* | 2022 | Association between preschooler movement behaviours, family dog ownership, dog play and dog walking: findings from the PLAYCE study | Preventive Medicine Reports | 10.1016/j.pmedr.2022.101753 | Article | Australia |

| **Authors** | **Year of publication** | **Title** | **Journal** | **Doi** | **Type of document** | **Country** |
| --- | --- | --- | --- | --- | --- | --- |
| Chuang *et al.* | 2010 | Catheter-related bacteremia caused by staphylococcus pseudintermedius refractory to antibiotic-lock therapy in a hemophilic child with dog exposure | Journal of Clinical Microbiology | 10.1128/JCM.02033-09 | Article | Taiwan |
| Cociancic *et al.* | 2018 | A cross-sectional study of intestinal parasitoses in dogs and children of the periurban area of La Plata (Buenos Aires, Argentina): zoonotic importance and implications in public health | Zoonoses and Public Health | 10.1111/zph.12408 | Article | Argentina |
| Collin *et al.* | 2015 | Pet ownership is associated with increased risk of non-atopic asthma and reduced risk of atopy in childhood: findings from a UK birth cohort | Clinical and Experimental Allergy: Journal of the British Society for Allergy and Clinical Immunology | 10.1111/cea.12380 | Article | UK |
| Connell *et al.* | 2019 | Dogs in schools: the impact of specific human-dog interactions on reading ability in children aged 6 to 8 years | Anthrozoos | 10.1080/08927936.2019.1598654 | Article | Australia |

| **Authors** | **Year of publication** | **Title** | **Journal** | **Doi** | **Type of document** | **Country** |
| --- | --- | --- | --- | --- | --- | --- |
| Crossman *et al.* | 2020 | The influence of interactions with dogs on affect, anxiety, and arousal in children | Journal of Clinical Child and Adolescent Psychology | 10.1080/15374416.2018.1520119 | Article | USA |
| Daniels *et al.* | 2009 | Analysis of nonfatal dog bites in children | Journal of Trauma - Injury, Infection and Critical Care | 10.1097/TA.0b013e3181937925 | Article | USA |
| Dankner *et al.* | 1987 | DF-2 bacteremia following a dog bite in a 4-month-old child | Pediatric Infectious Disease Journal | 10.1097/00006454-198707000-00020 | Article | USA |
| Davis | 1987 | Pet care during preadolescence: developmental considerations | Child: Care, Health and Development | 10.1111/j.1365-2214.1987.tb00543.x | Article | Canada |
| Davis *et al.* | 2012 | Dog bite risk: an assessment of child temperament and child-dog interactions | International Journal of Environmental Research and Public Health | 10.3390/ijerph9083002 | Article | Several |
| de Carvalho *et al.* | 2012 | Comminuted mandibular fracture in child victim of dog bite | Dental Traumatology | 10.1111/j.1600-9657.2011.01079.x | Article | Brazil |
| Demirbas *et al.* | 2016 | Adults' ability to interpret canine body language during a dog-child interaction | Anthrozoos | 10.1080/08927936.2016.1228750 | Article | Turkey |
| Dixon *et al.* | 2012 | Dog bite prevention: an assessment of child knowledge | Journal of Pediatrics | 10.1016/j.jpeds.2011.07.016 | Article | USA |

| **Authors** | **Year of publication** | **Title** | **Journal** | **Doi** | **Type of document** | **Country** |
| --- | --- | --- | --- | --- | --- | --- |
| Dixon *et al.* | 2020 | Dog bites in children surge during coronavirus disease-2019: a case for enhanced prevention | The Journal of Pediatrics | 10.1016/j.jpeds.2020.06.071 | Article | USA |
| Doogan *et al.* | 1992 | Origins of fear of dogs in adults and children: the role of conditioning processes and prior familiarity with dogs | Behaviour Research and Therapy | 10.1016/0005-7967(92)90050-Q | Article | UK |
| Dovgan *et al.* | 2020 | Dog phobia intervention: a case study in improvement of physiological and behavioral symptoms in a child with intellectual disability | Developmental Neurorehabilitation | 10.1080/17518423.2019.1683909 | Article | USA |
| Dueñas *et al.* | 2021 | The relationship between living with dogs and social and emotional development in childhood | Anthrozoos | 10.1080/08927936.2021.1878680 | Article | Spain |
| Duperrex *et al.* | 2009 | Education of children and adolescents for the prevention of dog bite injuries | Cochrane Database of Systematic Reviews | 10.1002/14651858.CD004726.pub2 | Review | World |
| Duteille *et al.* | 2002 | Tragic case of a dog bite in a young child: the dog stands trial | Annals of Plastic Surgery | 10.1097/00000637-200202000-00012 | Article | France |

| **Authors** | **Year of publication** | **Title** | **Journal** | **Doi** | **Type of document** | **Country** |
| --- | --- | --- | --- | --- | --- | --- |
| Dwyer *et al.* | 2007 | Dog bite injuries in children - A review of data from a South African paediatric trauma unit | South African Medical Journal | NA | Article | South Africa |
| Edner *et al.* | 2021 | Low risk of transmission of pathogenic bacteria between children and the assistance dog during animal-assisted therapy if strict rules are followed | Journal of Hospital Infection | 10.1016/j.jhin.2021.04.025 | Article | Sweden |
| Elmacı *et al.* | 2015 | Dog-assisted therapies and activities in rehabilitation of children with cerebral palsy and physical and mental disabilities | International Journal of Environmental Research and Public Health | 10.3390/ijerph120505046 | Article | Turkey |
| Endo *et al.* | 2020 | Dog and cat ownership predicts adolescents' mental well-being: A population-based longitudinal study | International Journal of Environmental Research and Public Health | 10.3390/ijerph17030884 | Article | Japan |
| Engelberg *et al.* | 2016 | Dog walking among adolescents: correlates and contribution to physical activity | Preventive Medicine | 10.1016/j.ypmed.2015.11.011 | Article | USA |
| Eppley *et al.* | 2013 | Facial dog bite injuries in children: treatment and outcome assessment | Journal of Craniofacial Surgery | 10.1097/SCS.0b013e31827fee33 | Article | USA |

| **Authors** | **Year of publication** | **Title** | **Journal** | **Doi** | **Type of document** | **Country** |
| --- | --- | --- | --- | --- | --- | --- |
| Epstein *et al.* | 2011 | Opposing effects of cat and dog ownership and allergic sensitization on eczema in an atopic birth cohort | The Journal of Pediatrics | 10.1016/j.jpeds.2010.07.026 | Article | USA |
| Eretová *et al.* | 2020 | Can children of different ages recognize dog communication signals in different situations? | International Journal of Environmental Research and Public Health | 10.3390/ijerph17020506 | Article | Czech Republic |
| Esposito *et al.* | 2013 | Dog and cat bite-associated infections in children | European Journal of Clinical Microbiology and Infectious Diseases | 10.1007/s10096-013-1840-x | Review | World |
| Etheredge *et al.* | 2004 | The roles of cats and dogs in the transmission of Toxoplasma infection in Kuna and Embera children in Eastern Panama | Pan American Journal of Public Health | 10.1590/s1020-49892004000900004 | Article | Panama |
| Fall *et al.* | 2015 | Early exposure to dogs and farm animals and the risk of childhood asthma | JAMA Pediatrics | 10.1001/jamapediatrics.2015.3219 | Article | Sweden |
| Fall *et al.* | 2018 | Dog characteristics and future risk of asthma in children growing up with dogs | Scientific Reports | 10.1038/s41598-018-35245-2 | Article | Sweden |
| Farnworth *et al.* | 2012 | Incidence of dog bites and public attitudes towards dog care and management in Samoa | Animal Welfare | 10.7120/09627286.21.4.477 | Article | Samoa |

| **Authors** | **Year of publication** | **Title** | **Journal** | **Doi** | **Type of document** | **Country** |
| --- | --- | --- | --- | --- | --- | --- |
| Farrell *et al.* | 2018 | Play-modified one-session treatment for young children with a specific phobia of dogs: a multiple baseline case series | Child Psychiatry and Human Development | 10.1007/s10578-017-0752-x | Article | Australia |
| Farrell *et al.* | 2021 | Virtual reality one-session treatment of child-specific phobia of dogs: a controlled, multiple baseline case series | Behavior Therapy | 10.1016/j.beth.2020.06.003 | Article | USA |
| Fein *et al.* | 2019 | Pediatric dog bites: a population-based profile | Injury Prevention | 10.1136/injuryprev-2017-042621 | Article | USA |
| Filiâtre *et al.* | 1990 | An experimental analysis of olfactory cues in child-dog interaction | Chemical Senses | 10.1093/chemse/15.6.679 | Article | France |
| Fung | 2015 | Increasing the social communication of a boy with autism using animal-assisted play therapy: a case report | Advances in mind-body medicine | NA | Article | Hong Kong |
| Furnell *et al.* | 2015 | Reducing dog bites in children | Community Practitioner | NA | Article | World |
| Gadomski *et al.* | 2015 | Pet dogs and children's health: opportunities for chronic disease prevention? | Preventing Chronic Disease | 10.5888/pcd12.150204 | Article | USA |

| **Authors** | **Year of publication** | **Title** | **Journal** | **Doi** | **Type of document** | **Country** |
| --- | --- | --- | --- | --- | --- | --- |
| Gadomski *et al.* | 2017 | Pet dogs and child physical activity: the role of child-dog attachment | Pediatric Obesity | 10.1111/ijpo.12156 | Article | USA |
| Gandhi *et al.* | 1999 | Dog bite injuries in children: a preliminary survey | American Surgeon | NA | Article | USA |
| Gee *et al.* | 2007 | The role of therapy dogs in speed and accuracy to complete motor skills tasks for preschool children | Anthrozoos | 10.2752/089279307X245509 | Article | USA |
| Gee *et al.* | 2010 | Preschool children require fewer instructional prompts to perform a memory task in the presence of a dog | Anthrozoos | 10.2752/175303710X12682332910051 | Article | USA |
| Gee *et al.* | 2012 | The presence of a therapy dog results in improved object recognition performance in preschool children | Anthrozoos | 10.2752/175303712X13403555186172 | Article | USA |
| Gee *et al.* | 2017 | Human–animal interaction research in school settings: current knowledge and future directions | AERA Open | 10.1177/2332858417724346 | Review | World |

| **Authors** | **Year of publication** | **Title** | **Journal** | **Doi** | **Type of document** | **Country** |
| --- | --- | --- | --- | --- | --- | --- |
| Georges *et al.* | 2008 | An investigation into the prevalence of dog bites to primary school children in Trinidad | BMC Public Health | 10.1186/1471-2458-8-85 | Article | Trinidad |
| Gola *et al.* | 2016 | Ethical considerations in exposure therapy with children | Cognitive and Behavioral Practice | 10.1016/j.cbpra.2015.04.003 | Review | World |
| Gouin *et al.* | 2021 | Description and determinants of at-risk interactions for human health between children and dogs in an Inuit village | Anthrozoos | 10.1080/08927936.2021.1926713 | Article | Canada |
| Gravrok *et al.* | 2018 | Adapting the traditional guide dog model to enable vision-impaired adolescents to thrive | Journal of Veterinary Behavior | NA | Article | Australia |
| Griffioen *et al.* | 2020 | Changes in behavioural synchrony during dog-assisted therapy for children with autism spectrum disorder and children with Down syndrome | Journal of Applied Research in Intellectual Disabilities | 10.1111/jar.12682 | Article | Netherlands |
| Grové *et al.* | 2021 | Therapy dogs in educational settings: guidelines and recommendations for implementation | Frontiers in Veterinary Science | 10.3389/fvets.2021.655104 | Article | Australia |

| **Authors** | **Year of publication** | **Title** | **Journal** | **Doi** | **Type of document** | **Country** |
| --- | --- | --- | --- | --- | --- | --- |
| Guillon *et al.* | 2014 | Both dog and human faces are explored abnormally by young children with autism spectrum disorders | NeuroReport | 10.1097/WNR.0000000000000257 | Article | France |
| Gullone *et al.* | 2008 | The relationship between bullying and animal abuse behaviors in adolescents: the importance of witnessing animal abuse | Journal of Applied Developmental Psychology | 10.1016/j.appdev.2008.06.004 | Article | Australia |
| Hall *et al.* | 2016 | Behavioral and self-report measures influencing children's reported attachment to their dog | Anthrozoos | 10.1080/08927936.2015.1088683 | Article | USA |
| Hall *et al.* | 2016 | Children reading to dogs: A systematic review of the literature | PLoS ONE | 10.1371/journal.pone.0149759 | Review | World |
| Hall *et al.* | 2016 | What factors are associated with positive effects of dog ownership in families with children with autism spectrum disorder? The development of the Lincoln autism pet dog impact scale | PLoS ONE | 10.1371/journal.pone.0149736 | Article | UK |

| **Authors** | **Year of publication** | **Title** | **Journal** | **Doi** | **Type of document** | **Country** |
| --- | --- | --- | --- | --- | --- | --- |
| Hall *et al.* | 2017 | Parent perceptions of the quality of life of pet dogs living with neuro-typically developing and neuro-atypically developing children: an exploratory study | PLoS ONE | 10.1371/journal.pone.0185300 | Article | UK |
| Hall *et al.* | 2019 | A systematic scoping review: what is the risk from child-dog interactions to dog's quality of life? | Journal of Veterinary Behavior | 10.1016/j.jveb.2019.05.001 | Review | World |
| Hall *et al.* | 2019 | Developing and assessing the validity of a scale to assess pet dog quality of life: Lincoln P-Qol | Frontiers in Veterinary Science | 10.3389/fvets.2019.00326 | Article | UK |
| Hamilton *et al.* | 1991 | Reliability of a Behavioral Avoidance Test for the assessment of dog phobic children | Psychological reports | 10.2466/pr0.1991.69.1.18 | Article | Australia |
| Hassan *et al.* | 2022 | A systematic review of dog-assisted therapy in children with behavioural and developmental disorders | Advances in Neurodevelopmental Disorders | 10.1007/s41252-022-00239-9 | Review | World |

| **Authors** | **Year of publication** | **Title** | **Journal** | **Doi** | **Type of document** | **Country** |
| --- | --- | --- | --- | --- | --- | --- |
| Hassan *et al.* | 2022 | Prevalence and associated factors of dog bites: a cross-sectional study among primary school children in Madawaki District Gusau, Nigeria | Malaysian Journal of Medicine and Health Sciences | NA | Article | Nigeria |
| Hawkins *et al.* | 2017 | Childhood attachment to pets: associations between pet attachment, attitudes to animals, compassion, and humane behaviour | International Journal of Environmental Research and Public Health | 10.3390/ijerph14050490 | Article | UK |
| Hediger *et al.* | 2020 | Dog-assisted therapy in neurorehabilitation of children with severe neurological impairment: an explorative study | Neuropediatrics | 10.1055/s-0040-1708545 | Article | Germany |
| Heimlich | 2001 | Animal-assisted therapy and the severely disabled child: a quantitative study | Journal of Rehabilitation | NA | Article | USA |
| Heljakka *et al.* | 2021 | A model for enhancing emotional literacy through playful learning with a robot dog | NA | 10.1109/ICECCME52200.2021.9590996 | Conference paper | Finland |
| Henderson *et al.* | 2020 | . An evaluation of a dog-assisted reading program to support student wellbeing in primary school | Children and Youth Services Review | 10.1016/j.childyouth.2020.105449 | Article | Australia |

| **Authors** | **Year of publication** | **Title** | **Journal** | **Doi** | **Type of document** | **Country** |
| --- | --- | --- | --- | --- | --- | --- |
| Hölscher *et al.* | 2002 | Exposure to pets and allergies in children | Pediatric Allergy and Immunology | 10.1034/j.1399-3038.2002.02063.x | Article | Germany |
| Horswell *et al.* | 2011 | Dog bites of the face, head and neck in children | The West Virginia Medical Journal | NA | Article | USA |
| Hull *et al.* | 2022 | Canine comfort: the protective effects of dog ownership and support for victimized adolescents | Anthrozoos | 10.1080/08927936.2022.2027092 | Article | USA |
| Hurst *et al.* | 2020 | Children have an increased risk of periorbital dog bite injuries | Journal of Oral and Maxillofacial Surgery | 10.1016/j.joms.2019.08.021 | Article | USA |
| Iliopoulou *et al.* | 2017 | Development of a Canine Care and Welfare Knowledge Scale: implications for welfare and preventing dogfighting | Journal of Applied Animal Welfare Science | 10.1080/10888705.2017.1330154 | Article | USA |
| Isparta *et al.* | 2021 | The first assessment of a dog bite prevention program for pre-school children in Turkey | Journal of Veterinary Behavior | 10.1016/j.jveb.2021.05.008 | Article | Turkey |
| Jakeman *et al.* | 2020 | Pet dog bites in children: management and prevention | BMJ Paediatrics Open | 10.1136/bmjpo-2020-000726 | Review | World |

| **Authors** | **Year of publication** | **Title** | **Journal** | **Doi** | **Type of document** | **Country** |
| --- | --- | --- | --- | --- | --- | --- |
| Jalongo | 2015 | An attachment perspective on the child-dog bond: interdisciplinary and international research findings | Early Childhood Education Journal | 10.1007/s10643-015-0687-4 | Review | World |
| Jalongo | 2018 | Keeping children safe: children’s ability to interpret canine behavioral cues and dog safety interventions | NA | 10.1007/978-3-319-77845-7_13 | Book chapter | World |
| Jalongo *et al.* | 2022 | Facility dogs in educational programs for young children: definition, rationale, issues, and implementation | Early Childhood Education Journal | 10.1007/s10643-022-01334-1 | Review | World |
| Jarolmen | 1998 | A companion of the grief reaction of children and adults: focusing on pet loss and bereavement | *OMEGA - Journal of Death and Dying* | 10.2190/H937-U230-X7D9-CVKH | Article | USA |
| Ji *et al.* | 2010 | Investigation of posttraumatic stress disorder in children after animal-induced injury in China | Pediatrics | 10.1542/peds.2009-3530 | Article | China |

| **Authors** | **Year of publication** | **Title** | **Journal** | **Doi** | **Type of document** | **Country** |
| --- | --- | --- | --- | --- | --- | --- |
| Jones *et al.* | 2019 | Incorporating animal-assisted therapy in mental health treatments for adolescents: a systematic review of canine assisted psychotherapy | PLoS ONE | 10.1371/journal.pone.0210761 | Review | World |
| Juríčková *et al.* | 2020 | Effect of animal assisted education with a dog within children with ADHD in the classroom: a case study | Child and Adolescent Social Work Journal | 10.1007/s10560-020-00716-x | Article | Czech Republic |
| Käck *et al.* | 2018 | Molecular allergy diagnostics refine characterization of children sensitized to dog dander | Journal of Allergy and Clinical Immunology | 10.1016/j.jaci.2018.05.012 | Article | Sweden |
| Kahn *et al.* | 2003 | Child victims of dog bites treated in emergency departments: a prospective survey | European Journal of Pediatrics | 10.1007/s00431-002-1130-6 | Article | Belgium |
| Kato *et al.* | 2003 | Survey of the stray dog population and the health education program on the prevention of dog bites and dog-acquired infections: a comparative study in Nepal and Okayama prefecture, Japan | Acta Medica Okayama | 10.18926/AMO/32829 | Article | Several |

| **Authors** | **Year of publication** | **Title** | **Journal** | **Doi** | **Type of document** | **Country** |
| --- | --- | --- | --- | --- | --- | --- |
| Kerepesi *et al.* | 2006 | Behavioural comparison of human-animal (dog) and human-robot (AIBO) interactions | Behavioural Processes | 10.1016/j.beproc.2006.04.001 | Article | Hungary |
| Kerns *et al.* | 2018 | Pet dogs: does their presence influence preadolescents’ emotional responses to a social stressor? | Social Development | 10.1111/sode.12246 | Article | USA |
| Kertes *et al.* | 2017 | Effect of pet dogs on children’s perceived stress and cortisol stress response: stress buffering effect of pet dogs | Social Development | 10.1111/sode.12203 | Article | USA |
| Kienesberger *et al.* | 2022 | Child safety programs for primary school children decrease the injury severity of dog bites | European Journal of Pediatrics | 10.1007/s00431-021-04256-z | Article | Austria |
| King *et al.* | 1997 | The etiology of childhood dog phobia | Behaviour Research and Therapy | 10.1016/s0005-7967(96)00067-8 | Review | World |
| Kirnan *et al.* | 2020 | A dog-assisted reading programme's unanticipated impact in a special education classroom | Educational Review | 10.1080/00131911.2018.1495181 | Article | USA |

| **Authors** | **Year of publication** | **Title** | **Journal** | **Doi** | **Type of document** | **Country** |
| --- | --- | --- | --- | --- | --- | --- |
| Krause-Parello *et al.* | 2018 | Examining the Effects of a Service-Trained Facility Dog on Stress in Children Undergoing Forensic Interview for Allegations of Child Sexual Abuse | Journal of Child Sexual Abuse | 10.1080/10538712.2018.1443303 | Article | USA |
| Kroten *et al.* | 2018 | Toxocarosis in children: poor hygiene habits and contact with dogs is related to longer treatment | Parasitology Research | 10.1007/s00436-018-5833-7 | Article | Poland |
| Lackmann *et al.* | 1992 | Surgical treatment of facial dog bite injuries in children | Journal of Cranio-Maxillofacial Surgery | 10.1016/s1010-5182(05)80472-x | Article | Germany |
| Lakestani *et al.* | 2014 | Interpretation of dog behavior by children and young adults | Anthrozoos | 10.2752/175303714X13837396326413 | Article | UK |
| Lakestani *et al.* | 2015 | Dog bite prevention: effect of a short educational intervention for preschool children | PLoS ONE | 10.1371/journal.pone.0134319 | Article | UK |
| Lang *et al.* | 2005 | Dog bites in Canadian children: a five-year review of severity and emergency department management | Canadian Journal of Emergency Medicine | 10.1017/s1481803500014494 | Article | Canada |

| **Authors** | **Year of publication** | **Title** | **Journal** | **Doi** | **Type of document** | **Country** |
| --- | --- | --- | --- | --- | --- | --- |
| Lange *et al.* | 2007 | Is counseling going to the dogs? An exploratory study related to the inclusion of an animal in group counseling with adolescents | Journal of Creativity in Mental Health | 10.1300/J456v02n02_03 | Review | World |
| Lavín-Pérez *et al.* | 2021 | Effects of dog-assisted therapy in adolescents with eating disorders: a study protocol for a pilot controlled trial | Animals | 10.3390/ani11102784 | Article | Spain |
| Lenihan *et al.* | 2016 | Measuring the effects of reading assistance dogs on reading ability and attitudes in elementary schoolchildren | Journal of Research in Childhood Education | 10.1080/02568543.2016.1143896 | Article | USA |
| Leonardi *et al.* | 2017 | “You think you’re helping them, but they’re helping you too”: experiences of Scottish male young offenders participating in a dog training program | International Journal of Environmental Research and Public Health | 10.3390/ijerph14080945 | Article | Scotland |
| Leung *et al.* | 2005 | Penile dog bite in an adolescent | Advances in Therapy | 10.1007/BF02850083 | Article | Canada |
| Li *et al.* | 2021 | Management of pet allergies in children in China | Current Treatment Options in Allergy | 10.1007/s40521-021-00279-x | Article | China |

| **Authors** | **Year of publication** | **Title** | **Journal** | **Doi** | **Type of document** | **Country** |
| --- | --- | --- | --- | --- | --- | --- |
| Linder *et al.* | 2017 | Dog attachment and perceived social support in overweight/obese and healthy weight children | Preventive Medicine Reports | 10.1016/j.pmedr.2017.04.014 | Article | USA |
| Lindsay *et al.* | 2021 | The impact of service dogs on children, youth and their families: a systematic review | Disability and Health Journal | 10.1016/j.dhjo.2020.101012 | Review | World |
| Lødrup *et al.* | 2021 | Does pet ownership in infancy lead to asthma or allergy at school age? Pooled analysis of individual participant data from 11 European birth cohorts | PLoS ONE | 10.1371/journal.pone.0043214 | Article | Several |
| Love *et al.* | 2001 | How anticipating relationships between dogs and children can help prevent disasters | Journal of the American Veterinary Medical Association | 10.2460/javma.2001.219.446 | Review | World |
| Macedo *et al.* | 2016 | Reconstruction of face and scalp after dog bites in children | Revista do Colegio Brasileiro de Cirurgioes | 10.1590/0100-69912016006007 | Article | Brazil |
| Madden Ellsworth *et al.* | 2016 | Interaction with shelter dogs reduces negative affect of adolescents in substance use disorder treatment | Anthrozoos | 10.1080/08927936.2016.1152719 | Article | USA |

| **Authors** | **Year of publication** | **Title** | **Journal** | **Doi** | **Type of document** | **Country** |
| --- | --- | --- | --- | --- | --- | --- |
| Mader *et al.* | 1989 | Social acknowledegments for children with disabilities: effects of service dogs | Child development | 10.1111/j.1467-8624.1989.tb04023.x | Article | USA |
| Mandhane *et al.* | 2009 | Cats and dogs and the risk of atopy in childhood and adulthood | Journal of Allergy and Clinical Immunology | 10.1016/j.jaci.2009.06.038 | Article | New Zealand |
| Marsa-Sambola *et al.* | 2016 | The Short Attachment to Pets Scale (SAPS) for children and young people: development, psychometric qualities and demographic and health associations | Child Indicators Research | 10.1007/s12187-015-9303-9 | Article | UK |
| Martin *et al.* | 2015 | Not just "A walking the dog": dog walking and pet play and their association with recommended physical activity among adolescents | American Journal of Health Promotion | 10.4278/ajhp.130522-ARB-262 | Article | Australia |
| Mathews *et al.* | 1994 | A behavioral analysis of dog bites to children | Journal of Developmental and Behavioral Pediatrics | 10.1097/00004703-199402000-00008 | Review | World |
| May *et al.* | 2013 | Evidence-based behavioral treatment of dog phobia with young children: two case examples | Behavior Modification | 10.1177/0145445512458524 | Article | USA |

| **Authors** | **Year of publication** | **Title** | **Journal** | **Doi** | **Type of document** | **Country** |
| --- | --- | --- | --- | --- | --- | --- |
| McDonald *et al.* | 2018 | Animal cruelty among children in violent households: children’s explanations of their behavior | Journal of Family Violence | 10.1007/s10896-018-9970-7 | Article | USA |
| McHeik *et al.* | 2000 | Treatment of facial dog bite injuries in children: A retrospective study | Journal of Pediatric Surgery | 10.1053/jpsu.2000.0350580 | Article | France |
| Medjo *et al.* | 2013 | Association between pet-keeping and asthma in school children: pets and asthma | Pediatrics International | 10.1111/ped.12071 | Article | China |
| Meints *et al.* | 2018 | Teaching children and parents to understand dog signaling | Frontiers in Veterinary Science | 10.3389/fvets.2018.00257 | Article | UK |
| Melco *et al.* | 2020 | Investigation of physiological and behavioral responses in dogs participating in animal-assisted therapy with children diagnosed with attention-deficit hyperactivity disorder | Journal of Applied Animal Welfare Science | 10.1080/10888705.2018.1536979 | Article | USA |
| Melson *et al.* | 2005 | Robots as dogs? - Children's interactions with the robotic dog AIBO and a live Australian shepherd | NA | 10.1145/1056808.1056988 | Conference paper | USA |

| **Authors** | **Year of publication** | **Title** | **Journal** | **Doi** | **Type of document** | **Country** |
| --- | --- | --- | --- | --- | --- | --- |
| Messam *et al.* | 2008 | The human-canine environment: a risk factor for non-play bites? | Veterinary Journal | 10.1016/j.tvjl.2007.08.020 | Article | Several |
| Messam *et al.* | 2018 | Factors associated with bites to a child from a dog living in the same home: a bi-national comparison | Frontiers in Veterinary Science | 10.3389/fvets.2018.00066 | Article | Several |
| Millot | 1994 | Olfactory and visual cues in the interaction systems between dogs and children | Behavioural Processes | 10.1016/0376-6357(94)90065-5 | Article | France |
| Millot *et al.* | 1988 | Children and their pet dogs : how they communicate | Behavioural Processes | 10.1016/0376-6357(88)90046-0 | Article | France |
| Minatoya *et al.* | 2020 | Cat and dog ownership in early life and infant development: A prospective birth cohort study of Japan environment and children's study | International Journal of Environmental Research and Public Health | 10.3390/ijerph17010205 | Article | Japan |
| Mitchell *et al.* | 1993 | Familiarity and the rarity of deception: two theories and their relevance to play between dogs (*Canis familiaris*) and humans (*Homo* *sapiens*) | Journal of Comparative Psychology | 10.1037/0735-7036.107.3.291 | Article | USA |
| Mitchell *et al.* | 2003 | Dog bites of the scalp, face, and neck in children | Laryngoscope | 10.1097/00005537-200303000-00018 | Article | USA |

| **Authors** | **Year of publication** | **Title** | **Journal** | **Doi** | **Type of document** | **Country** |
| --- | --- | --- | --- | --- | --- | --- |
| Miyata *et al.* | 1999 | Dog-bite injuries to the breast in children: deformities to secondary sex characteristics and their repair in an extended follow-up | Annals of Plastic Surgery | 10.1097/00000637-199911000-00014 | Article | Japan |
| Morales-Romero *et al.* | 2020 | Asthma prevalence, but not allergic rhinitis nor atopic dermatitis, is associated to exposure to dogs in adolescents | Allergologia et Immunopathologia | 10.1016/j.aller.2019.04.008 | Article | Mexico |
| Morrongiello *et al.* | 2013 | Examining parents' behaviors and supervision of their children in the presence of an unfamiliar dog: does the Blue Dog intervention improve parent practices? | Accident Analysis and Prevention | 10.1016/j.aap.2013.02.005 | Article | Canada |
| Mueller *et al.* | 2021 | Null effects of therapy dog interaction on adolescent anxiety during a laboratory-based social evaluative stressor | Anxiety, Stress and Coping | 10.1080/10615806.2021.1892084 | Article | USA |
| Muldoon *et al.* | 2015 | ‘Mum cleaned it and I just played with it’: children’s perceptions of their roles and responsibilities in the care of family pets | Childhood | 10.1177/0907568214524457 | Article | UK |

| **Authors** | **Year of publication** | **Title** | **Journal** | **Doi** | **Type of document** | **Country** |
| --- | --- | --- | --- | --- | --- | --- |
| Muldoon *et al.* | 2016 | Exploring children’s perspectives on the welfare needs of pet animals | Anthrozoos | 10.1080/08927936.2016.1181359 | Article | UK |
| Muldoon *et al.* | 2019 | The nature and psychological impact of child/adolescent attachment to dogs compared with other companion animals | Society and Animals | 10.1163/15685306-12341579 | Review | World |
| Murray | 2017 | Examining evidence on dog bite injuries and their management in children | Nursing children and young people | 10.7748/ncyp.2017.e859 | Review | World |
| Murray *et al.* | 1983 | The frequency and severity of cat allergy vs. dog allergy in atopic children | The Journal of Allergy and Clinical Immunology | 10.1016/0091-6749(83)90522-5 | Article | Canada |
| Nagasawa *et al.* | 2009 | Attachment between humans and dogs | Japanese Psychological Research | 10.1111/j.1468-5884.2009.00402.x | Article | Japan |
| Nagengast *et al.* | 1997 | The effects of the presence of a companion animal on physiological arousal and behavioral distress in children during a physical examination | Journal of Pediatric Nursing | 10.1016/S0882-5963(97)80058-9 | Article | USA |
| Náhlík *et al.* | 2022 | How Parents Perceive the Potential Risk of a Child-Dog Interaction | International Journal of Environmental Research and Public Health | 10.3390/ijerph19010564 | Article | Czech Republic |

| **Authors** | **Year of publication** | **Title** | **Journal** | **Doi** | **Type of document** | **Country** |
| --- | --- | --- | --- | --- | --- | --- |
| Naidoo *et al.* | 2020 | First report of successful refashioning using the bracka technique after complete glans penile amputation from a dog bite injury in a child | Turkish Journal of Urology | 10.5152/tud.2020.20142 | Article | South Africa |
| Ng *et al.* | 2021 | A study protocol for a randomised controlled trial to evaluate the effectiveness of a dog-facilitated physical activity minimal intervention on young children's physical activity, health and development: the PLAYCE PAWS trial | BMC Public Health | 10.1186/s12889-020-10034-7 | Article | Australia |
| Nixon *et al.* | 1980 | Dog bite injuries to children. Potential rabies threat to Australia | Medical Journal of Australia | 10.5694/j.1326-5377.1980.tb134742.x | Article | Australia |
| Noble *et al.* | 2018 | A study into the impact of the Reading Education Assistance Dogs scheme on reading engagement and motivation to read among early years foundation-stage children | Education 3-13 | 10.1080/03004279.2016.1246587 | Article | UK |

| **Authors** | **Year of publication** | **Title** | **Journal** | **Doi** | **Type of document** | **Country** |
| --- | --- | --- | --- | --- | --- | --- |
| Ogi *et al.* | 2016 | The relationship between children and family dogs concerning their possible aggressive behavior: a pilot study using a questionnaire for parents | Dog Behavior | 10.4454/db.v2i1.28 | Article | Italy |
| Oginni *et al.* | 2002 | Facial dog bites in Southwestern Nigerian children: an analysis of eight cases | Tropical Doctor | 10.1177/004947550203200423 | Article | Nigeria |
| Ogundare *et al.* | 2017 | Pattern and outcome of dog bite injuries among children in Ado-Ekiti, Southwest Nigeria | Pan African Medical Journal | 10.11604/pamj.2017.27.81.7360 | Article | Nigeria |
| Overall *et al.* | 2001 | Dog bites to humans—demography, epidemiology, injury, and risk | Journal of the American Veterinary Medical Association | 10.2460/javma.2001.218.1923 | Review | World |
| Owen *et al.* | 2010 | Family dog ownership and levels of physical activity in childhood: findings from the child heart and health study in England | American Journal of Public Health | 10.2105/AJPH.2009.188193 | Article | UK |

| **Authors** | **Year of publication** | **Title** | **Journal** | **Doi** | **Type of document** | **Country** |
| --- | --- | --- | --- | --- | --- | --- |
| Packer *et al.* | 2021 | Pandemic puppies: characterising motivations and behaviours of UK owners who purchased puppies during the 2020 COVID-19 pandemic | Animals | 10.3390/ani11092500 | Article | UK |
| Pai *et al.* | 2018 | Survey of knowledge of school children towards the prevalence, severity, management of maxillofacial injuries, and rescue skills in the event of a dog bite | Journal of Indian Society of Pedodontics and Preventive Dentistry | 10.4103/JISPPD.JISPPD_1110_17 | Article | India |
| Parente *et al.* | 2021 | Consequences of COVID-19 lockdown on children and their pets: dangerous increase of dog bites among the paediatric population | Children | 10.3390/children8080620 | Article | Italy |
| Parish-Plass | 2008 | Animal-assisted therapy with children suffering from insecure attachment due to abuse and neglect: a method to lower the risk of intergenerational transmission of abuse? | Clinical Child Psychology and Psychiatry | 10.1177/1359104507086338 | Article | Isreal |

| **Authors** | **Year of publication** | **Title** | **Journal** | **Doi** | **Type of document** | **Country** |
| --- | --- | --- | --- | --- | --- | --- |
| Park *et al.* | 2019 | Dog-bite injuries in Korea and risk factors for significant dog-bite injuries: a 6-year cross-sectional study | PLoS ONE | 10.1371/journal.pone.0210541 | Article | South Korea |
| Patterson *et al.* | 2022 | Pediatric dog bite injuries in the USA: a systematic review | World Journal of Pediatric Surgery | 10.1136/wjps-2021-000281 | Article | USA |
| Perzanowski *et al.* | 2002 | Effect of cat and dog ownership on sensitization and development of asthma among preteenage children | American Journal of Respiratory and Critical Care Medicine | 10.1164/rccm.2201035 | Article | Sweden |
| Peters *et al.* | 2004 | Posttraumatic stress disorder after dog bites in children | Journal of Pediatrics | 10.1016/j.jpeds.2003.10.024 | Article | Belgium |
| Plant *et al.* | 2019 | “It’s a dog’s life”: culture, empathy, gender, and domestic violence predict animal abuse in adolescents—Implications for societal health | Journal of Interpersonal Violence | 10.1177/0886260516659655 | Article | UK |
| Pongrácz *et al.* | 2005 | Human listeners are able to classify dog (*Canis familiaris*) barks recorded in different situations | Journal of Comparative Psychology | 10.1037/0735-7036.119.2.136 | Article | Hungary |

| **Authors** | **Year of publication** | **Title** | **Journal** | **Doi** | **Type of document** | **Country** |
| --- | --- | --- | --- | --- | --- | --- |
| Pongrácz *et al.* | 2011 | Do children understand man's best friend? Classification of dog barks by pre-adolescents and adults | Applied Animal Behaviour Science | 10.1016/j.applanim.2011.09.005 | Article | Hungary |
| Prothmann *et al.* | 2005 | Analysis of child-dog play behavior in child psychiatry | Anthrozoos | 10.2752/089279305785594261 | Article | Germany |
| Prothmann *et al.* | 2009 | Preference for, and responsiveness to, people, dogs and objects in children with autism | Anthrozoos | 10.2752/175303709X434185 | Article | Germany |
| Protopopova *et al.* | 2020 | Comparison of contingent and noncontingent access to therapy dogs during academic tasks in children with autism spectrum disorder | Journal of Applied Behavior Analysis | 10.1002/jaba.619 | Article | USA |
| Purewal *et al.* | 2017 | Companion animals and child/adolescent development: a systematic review of the evidence | International Journal of Environmental Research and Public Health | 10.3390/ijerph14030234 | Review | World |
| Pyrhönen *et al.* | 2015 | Dog and cat exposure and respective pet allergy in early childhood | Pediatric Allergy and Immunology | 10.1111/pai.12369 | Article | Finland |

| **Authors** | **Year of publication** | **Title** | **Journal** | **Doi** | **Type of document** | **Country** |
| --- | --- | --- | --- | --- | --- | --- |
| Pyrhönen *et al.* | 2018 | Coincidence of pollen season with the first fetal trimester together with early pet exposure is associated with sensitization to cat and dog allergens in early childhood: a Finnish population-based study | Clinical and Experimental Allergy | 10.1111/cea.13067 | Article | Finland |
| Racca *et al.* | 2012 | Reading faces: differential lateral gaze bias in processing canine and human facial expressions in dogs and 4-year-old children | PLoS ONE | 10.1371/journal.pone.0036076 | Article | UK |
| Radtke *et al.* | 2022 | Bibliotherapy for specific phobias of dogs in young children: a pilot study | Journal of Child and Family Studies | 10.1007/s10826-022-02304-2 | Article | USA |
| Ramgopal *et al.* | 2021 | Pediatric patients with dog bites presenting to US children’s hospitals | Injury Epidemiology | 10.1186/s40621-021-00349-3 | Article | USA |
| Redman | 1995 | Genital dog bite injuries in infants and children | Clinical Pediatrics | 10.1177/000992289503400607 | Article | USA |
| Reed *et al.* | 2022 | Isolated globe rupture without concomitant eyelid laceration or orbital trauma following facial dog bite injury in a child | American Journal of Ophthalmology Case Reports | 10.1016/j.ajoc.2022.101381 | Article | USA |

| **Authors** | **Year of publication** | **Title** | **Journal** | **Doi** | **Type of document** | **Country** |
| --- | --- | --- | --- | --- | --- | --- |
| Reisner *et al.* | 2008 | Effects of gender and parental status on knowledge and attitudes of dog owners regarding dog aggression toward children | Journal of the American Veterinary Medical Association | 10.2460/javma.233.9.1412 | Article | USA |
| Reisner *et al.* | 2011 | Behavioural characteristics associated with dog bites to children presenting to an urban trauma centre | Injury Prevention | 10.1136/ip.2010.029868 | Article | USA |
| Rew | 2000 | Friends and pets as companions: strategies for coping with loneliness among homeless youth | Journal of Child and Adolescent Psychiatric Nursing | 10.1111/j.1744-6171.2000.tb00089.x | Article | USA |
| Ribi *et al.* | 2008 | Comparison of children's behavior toward Sony's robotic dog AIBO and a real dog: a pilot study | Anthrozoos | 10.2752/175303708X332053 | Article | Switzerland |
| Riegger *et al.* | 1990 | Prevention and amelioration of stress and consequences of interaction between children and dogs | Journal of the American Veterinary Medical Association | NA | Article | USA |

| **Authors** | **Year of publication** | **Title** | **Journal** | **Doi** | **Type of document** | **Country** |
| --- | --- | --- | --- | --- | --- | --- |
| Rincón *et al.* | 2021 | Effects of dog-assisted education on physical and communicative skills in children with severe and multiple disabilities: a pilot study | Animals | 10.3390/ani11061741 | Article | Spain |
| Roberts *et al.* | 2017 | The influence of family dog ownership and parental perceived built environment measures on children’s physical activity within the Washington, DC area | International Journal of Environmental Research and Public Health | 10.3390/ijerph14111398 | Article | USA |
| Rolain *et al.* | 2009 | Serological evidence of Bartonella vinsonii lymphadenopathies in a child bitten by a dog | Clinical Microbiology and Infection | 10.1111/j.1469-0691.2008.02197.x | Article | France |
| Rosado *et al.* | 2009 | A comprehensive study of dog bites in Spain, 1995-2004 | Veterinary Journal | 10.1016/j.tvjl.2008.02.002 | Article | Spain |
| Rosano *et al.* | 2021 | Co-sleeping between adolescents and their pets may not impact sleep quality | Clocks & Sleep | 10.3390/clockssleep3010001 | Article | Australia |

| **Authors** | **Year of publication** | **Title** | **Journal** | **Doi** | **Type of document** | **Country** |
| --- | --- | --- | --- | --- | --- | --- |
| Rossman *et al.* | 1997 | Symptomatology and adaptive functioning for children exposed to normative stressors, dog attack, and parental violence | Journal of the American Academy of Child and Adolescent Psychiatry | 10.1097/00004583-199708000-00016 | Article | USA |
| Rowe *et al.* | 2021 | The curious incident of the dog in the nighttime: the effects of pet-human co-sleeping and bedsharing on sleep dimensions of children and adolescents | Sleep Health | 10.1016/j.sleh.2021.02.007 | Article | Canada |
| Russell | 2017 | “Everything has to die one day:” children’s explorations of the meanings of death in human-animal-nature relationships | Environmental Education Research | 10.1080/13504622.2016.1144175 | Article | Canada |
| Sabhaney *et al.* | 2012 | Management of dog bites in children | Canadian Family Physician | NA | Review | World |
| Sadiq *et al.* | 2015 | Eyelid lacerations due to dog bite in children | Journal of Pediatric Ophthalmology and Strabismus | 10.3928/01913913-20150901-02 | Article | USA |
| Salmon *et al.* | 2010 | Dog ownership, dog walking, and children's and parents' physical activity | Research Quarterly for Exercise and Sport | 10.1080/02701367.2010.10599674 | Article | Australia |

| **Authors** | **Year of publication** | **Title** | **Journal** | **Doi** | **Type of document** | **Country** |
| --- | --- | --- | --- | --- | --- | --- |
| Sato *et al.* | 2019 | Pet ownership and children’s emotional expression: propensity score-matched analysis of longitudinal data from Japan | International Journal of Environmental Research and Public Health | 10.3390/ijerph16050758 | Article | Japan |
| Schalamon *et al.* | 2006 | Analysis of dog bites in children who are younger than 17 years | Pediatrics | 10.1542/peds.2005-1451 | Article | Austria |
| Schoos *et al.* | 2020 | Children monosensitized to Can f 5 show different reactions to male and female dog allergen extract provocation: a randomized controlled trial | Journal of Allergy and Clinical Immunology: In Practice | 10.1016/j.jaip.2019.12.012 | Article | Denmark |
| Schretzmayer *et al.* | 2017 | Minor immediate effects of a dog on children's reading performance and physiology | Frontiers in Veterinary Science | 10.3389/fvets.2017.00090 | Article | Austria |
| Schrieber *et al.* | 2014 | Transmission of *Streptococcus dysgalactiae* subsp. equisimilis between child and dog in an aboriginal Australian community | Zoonoses and Public Health | 10.1111/zph.12057 | Article | Australia |

| **Authors** | **Year of publication** | **Title** | **Journal** | **Doi** | **Type of document** | **Country** |
| --- | --- | --- | --- | --- | --- | --- |
| Schwebel *et al.* | 2012 | The blue dog: Evaluation of an interactive software program to teach young children how to interact safely with dogs | Journal of Pediatric Psychology | 10.1093/jpepsy/jsr102 | Article | Canada |
| Schwebel *et al.* | 2016 | Evaluating a website to teach children safety with dogs: A randomized controlled trial | International Journal of Environmental Research and Public Health | 10.3390/ijerph13121198 | Article | USA |
| Shen *et al.* | 2013 | A multi-site study on knowledge, attitudes, beliefs and practice of child-dog interactions in rural China | International Journal of Environmental Research and Public Health | 10.3390/ijerph10030950 | Article | China |
| Shen *et al.* | 2013 | Dog safety in rural China: Children's sources of safety information and effect on knowledge, attitudes, and practices | Accident Analysis and Prevention | 10.1016/j.aap.2013.05.014 | Article | China |
| Shen *et al.* | 2016 | A randomized trial evaluating child dog-bite prevention in rural China through video-based testimonials | Health Psychology | 10.1037/hea0000273 | Article | China |

| **Authors** | **Year of publication** | **Title** | **Journal** | **Doi** | **Type of document** | **Country** |
| --- | --- | --- | --- | --- | --- | --- |
| Shen *et al.* | 2017 | Systematic review: interventions to educate children about dog safety and prevent pediatric dog-bite injuries: a meta-analytic review | Journal of Pediatric Psychology | 10.1037/hea0000273 | Review | World |
| Shi *et al.* | 1988 | Thelazia callipaeda (Nematoda: Spirurida): Transmission by flies from dogs to children in Hubei, China | Transactions of the Royal Society of Tropical Medicine and Hygiene | 10.2460/javma.232.4.542 | Article | China |
| Shuler *et al.* | 2008 | Canine and human factors related to dog bite injuries | Journal of the American Veterinary Medical Association | 10.2460/javma.232.4.542 | Article | USA |
| Sikana *et al.* | 2021 | Dog ownership practices and responsibilities for children's health in terms of rabies control and prevention in rural communities in Tanzania | PLoS Neglected Tropical Diseases | 10.1371/journal.pntd.0009220 | Article | Tanzania |
| Silva *et al.* | 2011 | Can dogs prime autistic children for therapy? evidence from a single case study | Journal of Alternative and Complementary Medicine | 10.1089/acm.2010.0436 | Article | Portugal |

| **Authors** | **Year of publication** | **Title** | **Journal** | **Doi** | **Type of document** | **Country** |
| --- | --- | --- | --- | --- | --- | --- |
| Silva *et al.* | 2018 | Can dogs assist children with severe autism spectrum disorder in complying with challenging demands? An exploratory experiment with a live and a robotic dog | Journal of Alternative and Complementary Medicine | 10.1089/acm.2017.0254 | Article | Portugal |
| Silva *et al.* | 2021 | Humans’ ability to assess emotion in dog barks only slightly affected by their country of residence, a replication of Pongracz *et al.* (2005) in a Portuguese sample | Animal Behavior and Cognition | 10.26451/abc.08.02.02.2021 | Article | Portugal |
| Sirard *et al.* | 2011 | Dog ownership and adolescent physical activity | American Journal of Preventive Medicine | 10.1016/j.amepre.2010.11.007 | Article | USA |
| Speirs *et al.* | 2015 | Dog bites to the upper extremity in children | Journal of Paediatrics and Child Health | 10.1111/jpc.12948 | Article | USA |
| Sprod *et al.* | 2017 | What effect does participating in an assistance dog program have on the quality of life of children with autism spectrum disorders and their caregivers? A systematic review of current literature | Journal of Social Inclusion | 10.36251/josi.122 | Review | World |

| **Authors** | **Year of publication** | **Title** | **Journal** | **Doi** | **Type of document** | **Country** |
| --- | --- | --- | --- | --- | --- | --- |
| Sribnick *et al.* | 2016 | Dog bite injuries in children: clinical implications for head involvement | Trauma | 10.1177/1460408616631772 | Article | USA |
| Sterrett *et al.* | 1984 | DAE (Dog Assisted Education) | The Diabetes Educator | 10.1177/014572178401000305 | Article | USA |
| Svensson | 2014 | The impact of the animals on children’s learning and their development—A study of what children learn from and with pets: the example of dog and cat | Problems of Education in the 21st Century | 10.33225/pec/14.59.77 | Article | Poland |
| Tan *et al.* | 2004 | Sensitivities of three county health department surveillance systems for child-related dog bites: 261 Cases (2000) | Journal of the American Veterinary Medical Association | 10.2460/javma.2004.225.1680 | Article | USA |
| Taniguchi *et al.* | 2020 | Associations of dog and cat ownership with wheezing and asthma in children: pilot study of the Japan Environment and children's study | PLoS ONE | 10.1371/journal.pone.0232604 | Article | Japan |
| Thys *et al.* | 2021 | Perceptions and practices of dog ownership and rabies control at a human-wildlife domestic animal interface in South Africa | Anthrozoos | 10.1080/08927936.2021.1885146 | Article | South Africa |

| **Authors** | **Year of publication** | **Title** | **Journal** | **Doi** | **Type of document** | **Country** |
| --- | --- | --- | --- | --- | --- | --- |
| Timperio *et al.* | 2008 | Is dog ownership or dog walking associated with weight status in children and their parents? | Health Promotion Journal of Australia | 10.1071/he08060 | Article | UK |
| Tin | 2007 | Dog bite wounds in a child | Hong Kong Medical Journal | NA | Article | Hong Kong |
| Tseng | 2022 | Brief report: above and beyond safety: psychosocial and biobehavioral impact of autism-assistance dogs on autistic children and their families | Journal of Autism and Developmental Disorders | 10.1007/s10803-021-05410-0 | Article | USA |
| Tuggle *et al.* | 1993 | Dog bites in children | Journal of Pediatric Surgery | 10.1016/0022-3468(93)90695-H | Article | USA |
| Tulloch *et al.* | 2021 | Paediatric emergency department dog bite attendance during the COVID-19 pandemic: an audit at a tertiary children’s hospital | BMJ Paediatrics Open | 10.1136/bmjpo-2021-001040 | Article | UK |
| Tyner *et al.* | 2016 | Nice doggie! Contact desensitization plus reinforcement decreases dog phobias for children with autism | Behavior Analysis in Practice | 10.1007/s40617-016-0113-4 | Article | USA |

| **Authors** | **Year of publication** | **Title** | **Journal** | **Doi** | **Type of document** | **Country** |
| --- | --- | --- | --- | --- | --- | --- |
| Uccheddu *et al.* | 2018 | Assessing behavior and stress in two dogs during sessions of a reading-to-a-dog program for children with pervasive developmental disorders | Dog Behavior | 10.4454/db.v4i3.83 | Article | Italy |
| Uccheddu *et al.* | 2019 | The impacts of a reading-to-dog programme on attending and reading of nine children with autism spectrum disorders | Animals | 10.3390/ani9080491 | Article | Italy |
| Uppal *et al.* | 2011 | Incisor tooth in the nose: anecdotal sequel to dog bite in a 3-year-old child | Indian Journal of Dental Research | 10.4103/0970-9290.90313 | Article | India |
| Vagnoli *et al.* | 2015 | Can presence of a dog reduce pain and distress in children during venipuncture? | Pain Management Nursing | 10.1016/j.pmn.2014.04.004 | Article | Italy |
| Vanto *et al.* | 1982 | Dog serum albumin as an allergen. IgE, IgG and lymphocyte responses in dog dander-sensitive asthmatic children | International Archives of Allergy and Applied Immunology | 10.1159/000233192 | Article | Finland |

| **Authors** | **Year of publication** | **Title** | **Journal** | **Doi** | **Type of document** | **Country** |
| --- | --- | --- | --- | --- | --- | --- |
| Vanto *et al.* | 1983 | Dog hypersensitivity in asthmatic children. a clinical study with special reference to the relationship between the exposure to dogs and the occurrence of hypersensitivity symptoms | Acta Peadiatrica | 10.1111/j.1651-2227.1983.tb09773.x | Article | Finland |
| Viau *et al.* | 2010 | Effect of service dogs on salivary cortisol secretion in autistic children | Psychoneuroendocrinology | 10.1016/j.psyneuen.2010.02.004 | Article | Canada |
| Vidović *et al.* | 1999 | Pet ownership, type of pet and socio-emotional development of school children | Anthrozoos | 10.2752/089279399787000129 | Article | Croatia |
| Vincent *et al.* | 2020 | Therapy dog support in pediatric dentistry: a social welfare intervention for reducing anticipatory anxiety and situational fear in children | Child and Adolescent Social Work Journal | 10.1007/s10560-020-00701-4 | Article | USA |
| Voith | 1980 | Prognosis of treatment for aggressive behavior of dogs toward children | Modern Veterinary Practice | NA | Review | World |
| Voith | 1981 | An approach to ameliorating aggressive behavior of dogs toward children | Modern Veterinary Practice | NA | Review | World |

| **Authors** | **Year of publication** | **Title** | **Journal** | **Doi** | **Type of document** | **Country** |
| --- | --- | --- | --- | --- | --- | --- |
| Vučinić *et al.* | 2019 | Children are victims of dog bites due to irresponsible dog ownership, parenthood, and managers of school institutions in Serbia | Journal of Veterinary Behavior | 10.1016/j.jveb.2018.12.005 | Article | Serbia |
| Wang *et al.* | 2017 | *Canis familiaris* allergen Can f 6: expression, purification and analysis of B-cell epitopes in Chinese dog allergic children | Oncotarget | 10.18632/oncotarget.21822 | Article | China |
| Wanser *et al.* | 2020 | Considering family dog attachment bonds: do dog-parent attachments predict dog-child attachment outcomes in animal-assisted interventions? | Frontiers in Psychology | 10.3389/fpsyg.2020.566910 | Article | USA |
| Wanser *et al.* | 2021 | Dog-human behavioral synchronization: family dogs synchronize their behavior with child family members | Animal Cognition | 10.1007/s10071-020-01454-4 | Article | USA |
| Wass *et al.* | 1996 | Dog bites causing upper-limb fractures in children | Injury | 10.1016/0020-1383(96)00039-3 | Article | UK |

| **Authors** | **Year of publication** | **Title** | **Journal** | **Doi** | **Type of document** | **Country** |
| --- | --- | --- | --- | --- | --- | --- |
| Wedl *et al.* | 2015 | Children with avoidant or disorganized attachment relate differently to a dog and to humans during a socially stressful situation | Anthrozoos | 10.1080/08927936.2015.1070002 | Article | Germany |
| Wenden *et al.* | 2021 | The relationship between dog ownership, dog play, family dog walking, and pre-schooler social–emotional development: findings from the PLAYCE observational study | Pediatric Research | 10.1038/s41390-020-1007-2 | Article | Australia |
| Weiss *et al.* | 2009 | "I love this dog"- Children's emotional attachment to the robotic dog AIBO | International Journal of Social Robotics | 10.1007/s12369-009-0024-4 | Article | Austria |
| Wernroth *et al.* | 2017 | Dog exposure during the first year of life and type 1 diabetes in childhood | JAMA Pediatrics | 10.1001/jamapediatrics.2017.0585 | Article | Sweden |
| Westgarth *et al.* | 2012 | Is childhood obesity influenced by dog ownership? No cross-sectional or longitudinal evidence | Obesity Facts | 10.1159/000345963 | Article | UK |

| **Authors** | **Year of publication** | **Title** | **Journal** | **Doi** | **Type of document** | **Country** |
| --- | --- | --- | --- | --- | --- | --- |
| Westgarth *et al.* | 2013 | A cross-sectional study of frequency and factors associated with dog walking in 9-10 year old children in Liverpool, UK | BMC Public Health | 10.1186/1471-2458-13-822 | Article | UK |
| Westgarth *et al.* | 2013 | Pet ownership, dog types and attachment to pets in 9-10 year old children in Liverpool, UK | BMC Veterinary Research | 10.1186/1746-6148-9-102 | Article | UK |
| Westgarth *et al.* | 2017 | The association between dog ownership or dog walking and fitness or weight status in childhood | Pediatric Obesity | 10.1111/ijpo.12176 | Article | UK |
| Westgarth *et al.* | 2017 | A birth cohort analysis to study dog walking in adolescence shows no relationship with objectively measured physical activity | Frontiers in Veterinary Science | 10.3389/fvets.2017.00062 | Article | UK |
| Wohlfarth *et al.* | 2013 | Dogs motivate obese children for physical activity: key elements of a motivational theory of animal-assisted interventions | Frontiers in Psychology | 10.3389/fpsyg.2013.00796 | Article | Germany |
| Wilde *et al.* | 2001 | A severe dog bite in a small child. (How can it become an ethical dilemma?) | Journal of the Medical Association of Thailand | NA | Article | Thailand |

| **Authors** | **Year of publication** | **Title** | **Journal** | **Doi** | **Type of document** | **Country** |
| --- | --- | --- | --- | --- | --- | --- |
| Williams *et al.* | 2018 | Microvascular replantation following facial dog bites in children: systematic review and management algorithm | Annals of Plastic Surgery | 10.1097/SAP.0000000000001485 | Review | World |
| Wilson *et al.* | 2003 | Prevention of dog bites: evaluation of a brief educational intervention program for preschool children | Journal of Community Psychology | 10.1002/jcop.10038 | Article | Australia |
| Wiseman *et al.* | 1983 | Major dog attack injuries in children | Journal of Pediatric Surgery | 10.1016/s0022-3468(83)80353-4 | Article | Canada |
| Wolan-Nieroda *et al.* | 2021 | Effect of dog-assisted therapy on psychomotor development of children with intellectual disability | Children | 10.3390/children8010013 | Article | Poland |
| Wright *et al.* | 2015 | Pet dogs improve family functioning and reduce anxiety in children with autism spectrum disorder | Anthrozoos | 10.1080/08927936.2015.1070003 | Article | UK |
| Wright *et al.* | 2015 | Acquiring a pet dog significantly reduces stress of primary carers for children with autism spectrum disorder: a prospective case control study | Journal of Autism and Developmental Disorders | 10.1007/s10803-015-2418-5 | Article | UK |

| **Authors** | **Year of publication** | **Title** | **Journal** | **Doi** | **Type of document** | **Country** |
| --- | --- | --- | --- | --- | --- | --- |
| Wu *et al.* | 2011 | Primary repair of facial dog bite injuries in children" | Pediatric Emergency Care | 10.1097/PEC.0b013e31822c1112 | Article | USA |
| Wu *et al.* | 2014 | Cat, dog and house dust mite allergen levels on children's soft toys | Journal of Asthma | 10.3109/02770903.2013.843097 | Article | Taiwan |
| Xiao *et al.* | 2007 | Possible transmission of *Cryptosporidium canis* among children and a dog in a household | Journal of Clinical Microbiology | 10.1128/JCM.00503-07 | Article | Peru |
| Yam *et al.* | 2012 | Children, parents, and pets exercising together (CPET) randomised controlled trial: study rationale, design, and methods | BMC Public Health | 10.1186/1471-2458-13-1096 | Article | UK |
| Yolken *et al.* | 2019 | Exposure to household pet cats and dogs in childhood and risk of subsequent diagnosis of schizophrenia or bipolar disorder | PLoS ONE | 10.1371/journal.pone.0225320 | Article | USA |
| Zangari *et al.* | 2021 | Dog bite injuries in a tertiary care children's hospital: a seven-year review | Pediatrics International | 10.1111/ped.14484 | Article | Italy |

| **Authors** | **Year of publication** | **Title** | **Journal** | **Doi** | **Type of document** | **Country** |
| --- | --- | --- | --- | --- | --- | --- |
| Zhao *et al.* | 2006 | A comparative study of asthma, pollen, cat and dog allergy among pupils and allergen levels in schools in Taiyuan city, China, and Uppsala, Sweden | Indoor Air | 10.1111/j.1600-0668.2006.00433.x | Article | China |
| Zhu *et al.* | 2020 | Severe multiple rabid dog bite injuries in a child in central China: continuous 10-year observation and analysis on this case | Human Vaccines and Immunotherapeutics | 10.1080/21645515.2019.1676630 | Article | China |
| Zirngibl *et al.* | 2002 | Exposure to pets and atopic dermatitis during the first two years of life. a cohort study | Pediatric Allergy and Immunology | 10.1034/j.1399-3038.2002.01110.x | Article | Germany |
